# Diagnostic accuracy of gadoxetic acid-enhanced MR for small hypervascular hepatocellular carcinoma and the concordance rate of Liver Imaging Reporting and Data System (LI-RADS)

**DOI:** 10.1371/journal.pone.0178495

**Published:** 2017-05-30

**Authors:** Jae Seok Bae, Jung Hoon Kim, Mi Hye Yu, Dong Ho Lee, Hyo-Cheol Kim, Jin Wook Chung, Joon Koo Han

**Affiliations:** 1 Department of Radiology, Seoul National University Hospital, Seoul, Korea; 2 Institute of Radiation Medicine, Seoul National University College of Medicine, Seoul, Korea; Yonsei University College of Medicine, REPUBLIC OF KOREA

## Abstract

**Background & aims:**

To assess diagnostic accuracy of gadoxetic acid–enhanced MR for small hypervascular hepatocellular carcinoma (HCC) detected by C-arm CT and concordance rate of Liver Imaging Reporting and Data System (LI-RADS).

**Methods:**

In this retrospective study, we recruited 4,544 patients suspected of having HCC underwent C-arm CT from November 2008 to May 2013. Among these patients, gadoxetic acid–enhanced MR was performed in 167 patients with HCC (n = 379; 257 > 1 cm, 122 ≤ 1 cm). HCC was confirmed by MR, CT, or follow-up images. Two radiologists graded likelihood of HCC and assessed MR features. Jackknife alternative free-response receiver operating characteristic (JAFROC) analysis was performed. All HCCs were evaluated concordance rate of LI-RADS.

**Results:**

Mean JAFROC figure of merit for large (>1-cm) HCC was 0.948, while that for small HCC was 0.787 with fair agreement (κ = 0.409). Mean sensitivity and positive predictive value (PPV) were 91% and 90% for large HCC versus 63.0% and 79% for small HCC, respectively. Seventeen of 122 small HCCs (13.9%) were not visible on MR. Among 379 HCCs, 99 met LR-5, and 259 met LR-4. Common features for small HCC included arterial enhancement (81.9%), hepatobiliary phase hypointensity (80.3%), and delayed washout (72.9%).

**Conclusion:**

Diagnostic accuracy of gadoxetic acid–enhanced MR imaging for small, hypervascular HCCs (Mean figure of merit = 0.787) was still low compared with large HCC (Mean figure of merit = 0.948). LR-5 and LR-4 covered 94% (358/379) of the HCCs.

## Introduction

Hepatocellular carcinoma (HCC) accounts for 70% to 85% of the total number of hepatic malignancies [1]. As underlying chronic liver diseases are the most important risk factor for HCC, major international study groups, including the American Association for the Study of Liver Diseases (AASLD), the European Association for the Study of the Liver (EASL), and the Asian Pacific Association for the Study of the Liver (APASL), recommend ultrasound surveillance of these patients [2–4]. If a nodule detected on surveillance is ≥ 1 cm and shows typical arterial enhancement and portal/delayed washout on dynamic CT or MRI, the lesion is diagnosed as HCC without pathology confirmation. However, there is controversy regarding the management of small (< 1 cm) lesions. Repeated follow-up with ultrasound three to four months following the diagnosis is recommended in western countries, although the small hypervascular lesions could be diagnosed as HCCs according to the APASL guidelines [2–4]. It is difficult to diagnose small HCCs as the typical vascular kinetics of HCC is absent in small lesions and there are many mimickers of small HCC, such as dysplastic nodules or arterioportal shunts [5]. In addition, the confirmative diagnosis of small HCCs is often problematic due to the limitation of the standard of reference. As lesions smaller than the cutting interval could be overlooked in the pathology evaluation [6], this can result in their underestimation, especially in a retrospective study setting. Nevertheless, the low diagnostic performance reported in recent studies could be associated with these demonstrative features of small HCCs [7–9].

C-arm CT performed during trans-arterial chemoembolization (TACE) allows the detection of hypervascular tumor with high spatial resolution with a thin slice thickness [10]. In addition, the intra-arterial bolus injection of contrast medium for C-arm CT leads to better contrast-to-noise ratio, especially for hypervascular HCC, than that of CT and MRI [11, 12]. According to previously published reports, C-arm CT has been shown to have sufficient image quality for detecting HCCs [13–15]. Meanwhile, the Liver Imaging Reporting and Data System (LI-RADS) is a recently introduced, comprehensive system for reporting and interpreting CT or MR liver examinations performed for patients with a high risk of HCC [16]. However, there have only been a few reports regarding the concordance rate of HCCs, according to the LI-RADS. Therefore, we performed this study in order to evaluate the diagnostic accuracy and imaging features of gadoxetic acid-enhanced MRI for hypervascular HCC and with a referencing standard of C-arm CT. We also evaluated the concordance rate of the HCCs according to the LI-RADS.

## Materials and methods

### Study Population

**Fig 1:**
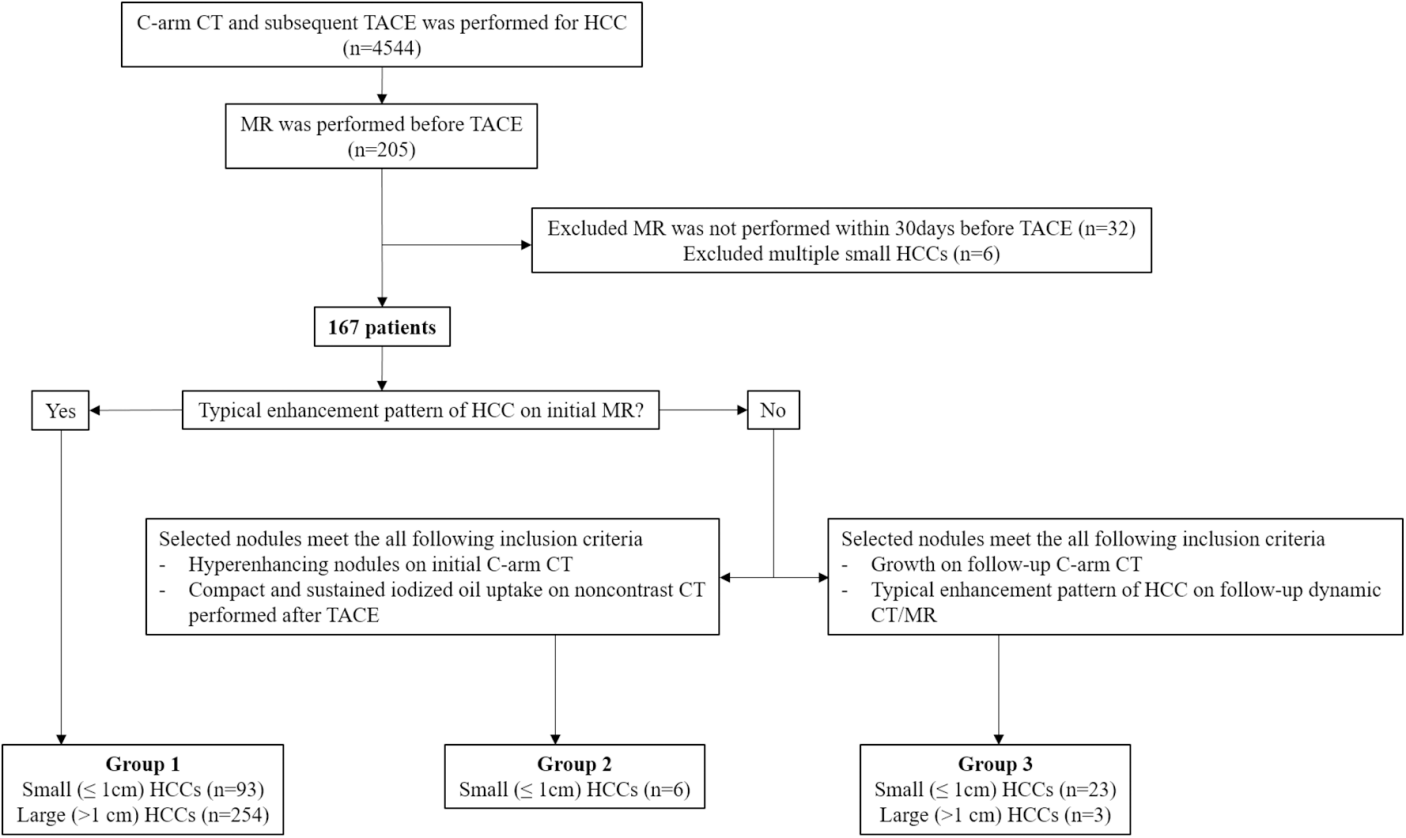
Flowchart of the study population.

This retrospective study was approved by the institutional review board of Seoul National University Hospital, and the informed consent was waived. From November 2008 to May 2013, 4,544 patients suspected of having HCC underwent C-arm CT-assisted TACE. Among these patients, 205 underwent gadoxetic acid–enhanced MRI before TACE. We excluded 38 patients in whom there were more than 30 days between the MR and TACE (n = 32) and those with more than 10 HCCs in a single patient (n = 6). Finally, 167 patients (mean age, 63.8 years ± 9.0; age range, 38–90 years) with 379 HCCs (257 HCCs > 1 cm, 122 HCCs ≤ 1 cm) were enrolled in our study, and the mean time interval between MRI and TACE was 12.02 days ± 8.60 (range, 0–30 days) (Fig 1). Among the patients, 33 of 167 patients have been previously reported [8]. For the control group, we searched our pathology and medical database from 2011 to 2012 and selected 66 patients who had undergone liver transplantation without HCC. We summarized the characteristics of patients and control group in **Table 1**.

**Table 1:** Clinical characteristics of patients and control group.

Characteristics	Patients (n = 122)	Control group (n = 66)
M:F	131:36	44:22
Mean age (age range)	63.8 ± 9.0 years (38–90 years)	57.2 ± 10.4 years (25–78 years)
Underlying disease		
Hepatitis virus B infection	126	28
Hepatitis virus C infection	24	10
Alcoholics	11	19
Non-B, non-C hepatitis	4	4
Non Alcoholic Fatty Liver Disease	2	0
Intrahepatic duct stone		2
Hepatitis virus A-associated acute hepatitis		1
Primary sclerosing cholangitis		1
Wilson's disease		1
Mean number of HCC (range)	2.27 ± 1.45 (1–8)	N/A
Mean interval between MR and TACE (date range)	12.02 ± 8.60 days (0–30 days)	N/A

Numbers in parentheses are ranges

### Diagnosis of HCC

Two radiologists (J.S.B. and J.H.K, with three and 18 years of clinical experience in abdominal imaging, respectively) carefully reviewed the initial MR images and C-arm CT images and categorized the HCCs into three groups based on the detection of HCC.

**Group 1** (254 HCCs > 1 cm, 93 HCCs ≤ 1 cm) referred to HCC diagnosed on the initial MR images. It was divided into two groups, including lesions > 1 cm and with the typical enhancement pattern of HCC, including arterial enhancement and portal/delayed washout seen on dynamic MR images and which were diagnosed as HCC according to the AASLD guidelines [2]. For the lesions ≤ 1 cm, two additional criteria were applied: (a) hyperenhancement seen on C-arm CT; and (b) dense, compact iodized oil uptake (Lipiodol; Andre Guerbet, Aulnay- Sous Bois, France) seen at follow-up CT. Retention of iodized oil after TACE is known to be highly predictive for neoplastic foci. Consequently we could consider the small lesions in Group 1 to be HCCs. **Group 2** (six HCCs ≤ 1 cm) referred to HCC diagnosed on the initial C-arm CT and follow-up CT. In this group, the nodules showed hyperenhancement on C-arm CT and, in addition, dense, compact iodized oil uptake on follow-up CT, even though the nodules did not show the typical enhancement pattern on the initial MRI. **Group 3** (three HCCs > 1 cm and 23 HCCs ≤ 1 cm) referred to the HCC diagnosed on follow-up C-arm CT and CT or MRI. This group included the nodules seen on the initial C-arm CT and observed to have enlarged on follow-up C-arm CT (mean time interval, 269.4 days ± 144.5; range, 75–809 days). At that time, the nodules showed the typical enhancement pattern of HCC on both CT and MRI.

### MR examination

MRI was performed on either a 1.5-T or 3-T superconducting system using either an eight-channel or a 32-channel, phased-array coil. 1.5-T machines were as follows: a Signa HDx [n = 80] or a Signa Excite [n = 1], GE Medical Systems, Milwaukee, WI, USA; Achieva [n = 5], a Intera [n = 1], Philips Medical Systems, Best, Netherlands). 3-T machines were as follows: Verio [n = 33], Trio [n = 24] or Avanto [n = 4], Siemens Medical Solutions, Erlangen, Germany; Signa Excite [n = 2], GE Medical Systems; Ingenia [n = 16] or Achieva [n = 2], Philips Medical Systems, Best, Netherlands. The routine liver MR imaging protocols performed at our hospital were as follows: a breath-hold, fat-saturated, T2-weighted, fast spin-echo or turbo spin-echo sequence; a breath-hold, T1-weighted, dual-echo (in-phase and opposed-phase) sequence; dynamic, three-dimensional, fat-saturated, T1-weighted sequences; and free-breathing, diffusion-weighted imaging (DWI) using a single-shot, echo-planar imaging sequence. Dynamic imaging was performed using three-dimensional, fat-saturated, T1-weighted sequences with a spatial resolution of 1.2–1.7 mm and a section thickness of 3–6 mm before and following administration of gadoxetic acid (Primovist; Bayer Healthcare, Berlin, Germany). Gadoxetic acid was injected intravenously as a bolus of 10 mL and at a rate of 1 or 1.5 mL/sec using a power injector (Spectris Solaris EP; Medrad, Warrendale, PA, USA), immediately followed by a 25-mL saline flush. Following the administration of contrast material, the imaging delay times were determined using real-time MR fluoroscopic monitoring. The arterial phase was imaged seven seconds after the contrast material arrived at the distal end of the thoracic aorta. Subsequently, portal venous phase, delayed phase, and HBP images were acquired 60 seconds, three minutes, 10 minutes, and 20 minutes, respectively, after injection of contrast material. Between the 10- and 20-minute delayed phase acquisitions, DWI with multiple b values (0 sec/mm2 and 500 sec/mm2) and simultaneous respiratory triggering were performed in the axial plane. The detailed parameters of the pulse sequences are listed in the **S1 Table.**

### C-Arm CT acquisition during TACE

TACE was performed in an interventional procedure unit equipped with a digital subtraction angiography device (AXIOM Artis dTA/VB30; Siemens). Two, clinically experienced interventional radiologists (000 with 17 years of clinical practice and 000 with 30 years of clinical practice) performed all of the angiographic examinations.

For C-arm CT acquisition, before infusion of a chemotherapeutic agent, a single series of three-dimensional, rotational, C-arm angiographic images of the common or proper hepatic artery was obtained for eight seconds during a single breath hold and with a 211° circular trajectory. The contrast agent (Iopamidol 300 mg as iodine, Pamiray 300; Dongkook Pharmaceutical, Seoul, South Korea) was injected at a flow rate of 2–4 mL/sec for 12 seconds using a power injector, and the images were acquired four seconds following the injection. The C-arm CT parameters were as follows: 512 × 512 matrix in projections; 211° total angle with approximately a 26° rotation per second; 0.5° increment; a system dose of approximately 0.36 mGy per frame; and a total of approximately 420 projections obtained. The obtained images were immediately transferred to a dedicated workstation (Leonardo with Dyna CT; Siemens Healthcare) which promptly reconstructed them with a section thickness of 0.4 mm. If there was an anatomic variation in the hepatic artery, such as the left hepatic artery coming from the left gastric artery and the right hepatic artery arising from the superior mesenteric artery, three-dimensional rotational C-arm angiographic images of the left and right hepatic arteries were obtained separately. As a result, the entire liver was fully covered in the scanning range in all patients [15, 17].

### Image analysis

Two, clinically experienced abdominal radiologists (M.H.Y. and D.H.L., both with 10 years of experience in abdominal imaging) independently analyzed the MR images in order to assess the diagnostic performance. The two radiologists were provided with readout sheets with a sample. They were informed that there was a control group and were blinded to the number, size, and locations of the lesions. Each observer recorded the size and segmental location of the hepatic lesions they identified. The reviewers added the image number in order to avoid any confusion when a patient had multiple lesions. On MR images, each reviewer evaluated the signal intensities on T1- and T2-weighted images as well as the arterial enhancement, signal intensities on the portal-phase images, three-minute delayed-phase images, and HBP images as well as the restriction seen on DWI. They then graded the possibility of HCC using a five-point confidence scale for each lesion as follows: 1, definitely benign lesion; 2, probably benign lesion; 3, indeterminate lesion; 4, probably HCC; and 5, definitely HCC. The diagnostic criteria for HCC were based on the MR imaging features, i.e. if a nodule showed arterial enhancement, portal or delayed washout or HBP hypointensity, it was rated with a score of 5. If a nodule showed no definite arterial enhancement but showed hypointensity on the portal, delayed or HBP images and there was an additional imaging feature among the hyperintensity seen on T2-weighted images or the hyperintensity on DWI, we rated it with a score of 4. If a nodule only showed hypointensity on the HBP images or had only arterial enhancement with a triangular or irregular shape, it was given a rating of 1 or 2, according to the subjective judgment.

We also analyzed the imaging features of HCCs and the concordance rate of the HCCs according to the LI-RADS. Concordance rate was the proportion of LI-RADS categories in our HCCs. For example, 26% of concordance rate for LI-RADS 5 meant that 26% of our HCCs belonged to LI-RADS category 5. Two, additional radiologists (000 and 000, with three and 17 years of clinical experience in abdominal imaging, respectively) who did not participate in the diagnostic performance study reviewed the MR images in consensus. They were given the information regarding the location and size of each HCC and for which they analyzed the major and ancillary imaging features of LI-RADS [18]. Finally, the concordance rate of each HCC to LI-RADS was evaluated. All image reviews were performed using picture archiving and communication system software (Infinitt, Seoul, South Korea) on a workstation computer (XW6200; Hewlett-Packard, Palo Alto, CA, USA) with monitors that had a spatial resolution of 1600 × 1200 (Totoku, Tokyo, Japan).

### Statistical analysis

We used Kruskal-Wallis test to compare size of the lesions among three Groups. To evaluate the diagnostic performance of MR imaging for detecting HCC, jackknife, alternative free-response, receiver-operating-characteristic (JAFROC) analysis was used with JAFROC software (JAFROC, version 4.2.1; http://www.devchakraborty.com) [19]. We calculated the mean diagnostic accuracy according to the mean figure of merit which was defined as the probability that on normal images, a lesion is rated higher than the highest rated non-lesion seen on control MR images of the liver [20]. We also calculated the sensitivity and positive predictive value (PPV) for detecting HCC on a per-lesion basis and on a per-patient basis. With regard to a per-patient analysis, the reviewer’s assessment was considered to be positive when he or she detected at least one lesion in the liver. The weighted κ value was used to evaluate the interobserver agreement of the confidence scale regarding the possibility of HCC detected on MR images. The scale for the κ coefficients for interobserver agreement was as follows: less than 0.20, poor; 0.21–0.40, fair; 0.41–0.60, moderate; 0.61–0.80, substantial; and 0.81–1.00, almost perfect. To evaluate statistical differences in the major and ancillary MR imaging features of small and large HCCs, the chi-square test and Fisher’s exact test were performed. The sensitivities and PPVs of the two groups were then assessed. A two-sided *P* value < .05 was considered to indicate statistical significance. Statistical analyses were carried out using a commercially available software package (SPSS version 22; SPSS, Chicago, IL, USA).

## Results

The mean size of all of the HCCs was 1.46 cm ± 0.8 (range, 0.2–10.5 cm). The mean size of small HCCs was 0.74 cm ± 0.21 (range, 0.2–1.0 cm) and that of large HCCs was 1.80 cm ± 0.78 (range, 1.1–10.5 cm). The size of the HCCs in group 1 (1.54 cm ± 0.81) was larger than that of the HCCs in group 2 (0.44 cm ± 0.23) and that of the HCCs in group 3 (0.64 cm ± 0.28) (*P* < .05).

**Fig 2:**
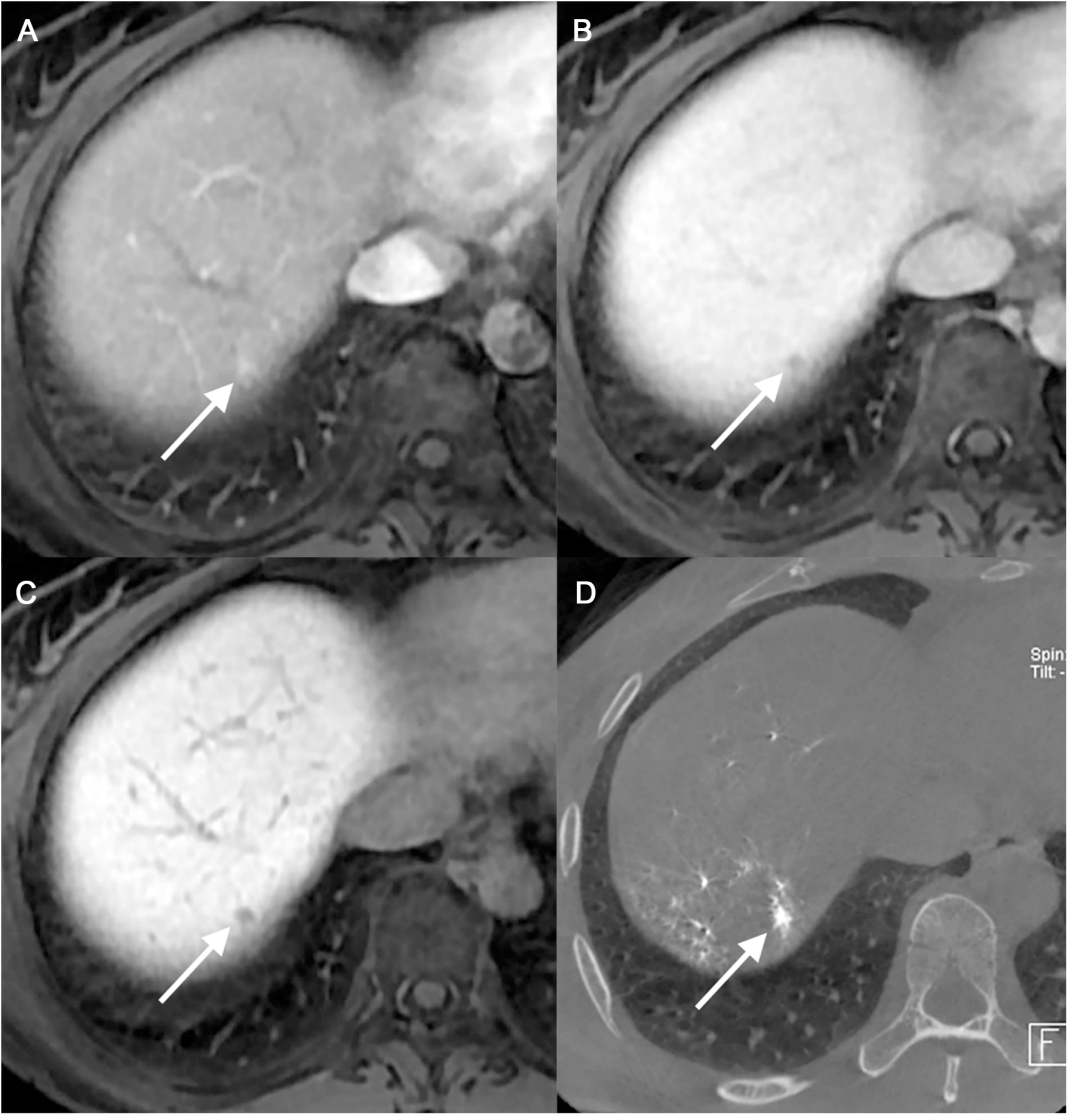
A 60-year-old woman with small hypervascular HCC (Group 1). (a-c) Initial MR images show a 5mm-sized nodule (arrow) in the segment 8 dome of the liver with arterial enhancement (a), washout on the 3-minute delayed phase image (b) and hypointensity on the HBP image (c). (d) This nodule demonstrated hyperenhancement at C-arm CT during TACE (d). This nodule showed continuous compact iodized oil uptake on follow-up CT (not shown). Both of the reviewers considered this lesion to be an HCC.

The diagnostic performance of MRI for HCCs on a per-lesion basis are summarized in **Table 2**. The figure of merit of small HCCs was 0.774 and 0.800 for reviewer 1 and reviewer 2, respectively, while the figure of merit for large HCCs was 0.963 and 0.932 for reviewer 1 and reviewer 2, respectively. The mean sensitivity and PPV for the detection of HCC were 62.7% (153 of 244) and 78.9% (153 of 194) in small HCCs and 90.7% (466 of 514) and 90.1% (466 of 517) in large HCCs, respectively (Fig 2**)**. In a per-patient analysis, the sensitivity and PPV for small HCCs were 56% (42 of 75) and 66.7% (42 of 63) for reviewer 1 and 54.7% (41 of 75) and 80.4% (41 of 51) for reviewer 2, respectively (**Table 3**). The interobserver agreement for the possibility of HCC was moderate (κ = 0.409).

**Table 2:** Diagnostic performance of gadoxetic acid–enhanced mr imaging for detection of HCC: Per-lesion analysis.

Parameter	Small (≤1-cm) HCC (n = 122)	Large (>1-cm) HCC (n = 257)	Total (n = 379)
Figure of merit			
Reviewer 1	0.774 (0.712, 0.836)	0.963 (0.946, 0.980)	0.921 (0.895, 0.946)
Reviewer 2	0.800 (0.744, 0.855)	0.932 (0.900, 0.963)	0.915 (0.888, 0.941)
Mean	0.787	0.948	0.918
Sensitivity (%)			
Reviewer 1	63 [77/122]	92 [236/257]	82 [313/379]
Reviewer 2	62 [76/122]	89 [230/257]	81 [306/379]
Mean	63 [153/244]	91 [466/514]	82 [619/758]
PPV (%)			
Reviewer 1	72 [77/107]	92 [236/257]	86 [313/364]
Reviewer 2	87 [76/87]	88 [230/260]	88 [306/347]
Mean	79 [153/194]	90 [466/517]	87 [619/713]

Note—Numbers in parentheses are 95% confidence intervals, and numbers in brackets are raw data.

**Table 3:** Diagnostic performance of gadoxetic acid–enhanced MR imaging for detection of HCC: Per-patient analysis.

Parameter	Small (≤1-cm) HCC (n = 75)	Large (>1-cm) HCC (n = 155)	Total (n = 167)
Sensitivity (%)			
Reviewer 1	56 [42/75]	88 [136/155]	71 [119/167]
Reviewer 2	55 [41/75]	83 [129/155]	69 [116/167]
PPV (%)			
Reviewer 1	67 [42/63]	89 [136/152]	82 [161/196]
Reviewer 2	80 [41/51]	85 [129/151]	84 [163/193]

Note—Numbers in parentheses are 95% confidence intervals, and numbers in brackets are raw data.

**Fig 3:**
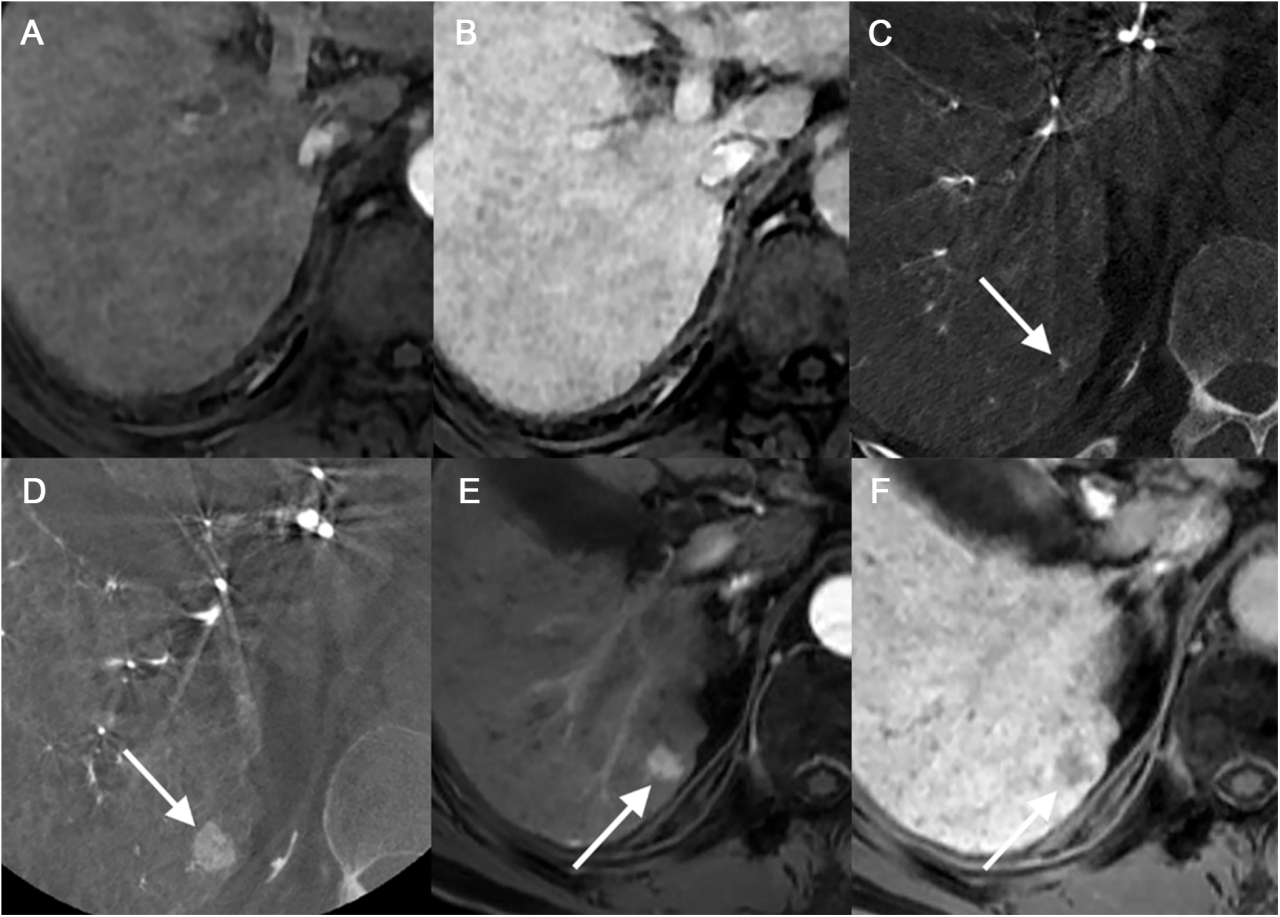
A 73-year-old man with a 2mm-sized small hypervascular HCC (Group 3). (a-c) This lesion was not detectable on the initial MR on either arterial phase (a) or hepatobiliary phase (b). However, it was noted as a 2mm enhancing lesion on initial C-arm CT (c). (d-f) After 13 months without treatment, this lesion showed growth to 13mm on follow-up C-arm CT (d), demonstrated arterial enhancement (e) and hepatobiliary phase hypointensity (f).

**Fig 4:**
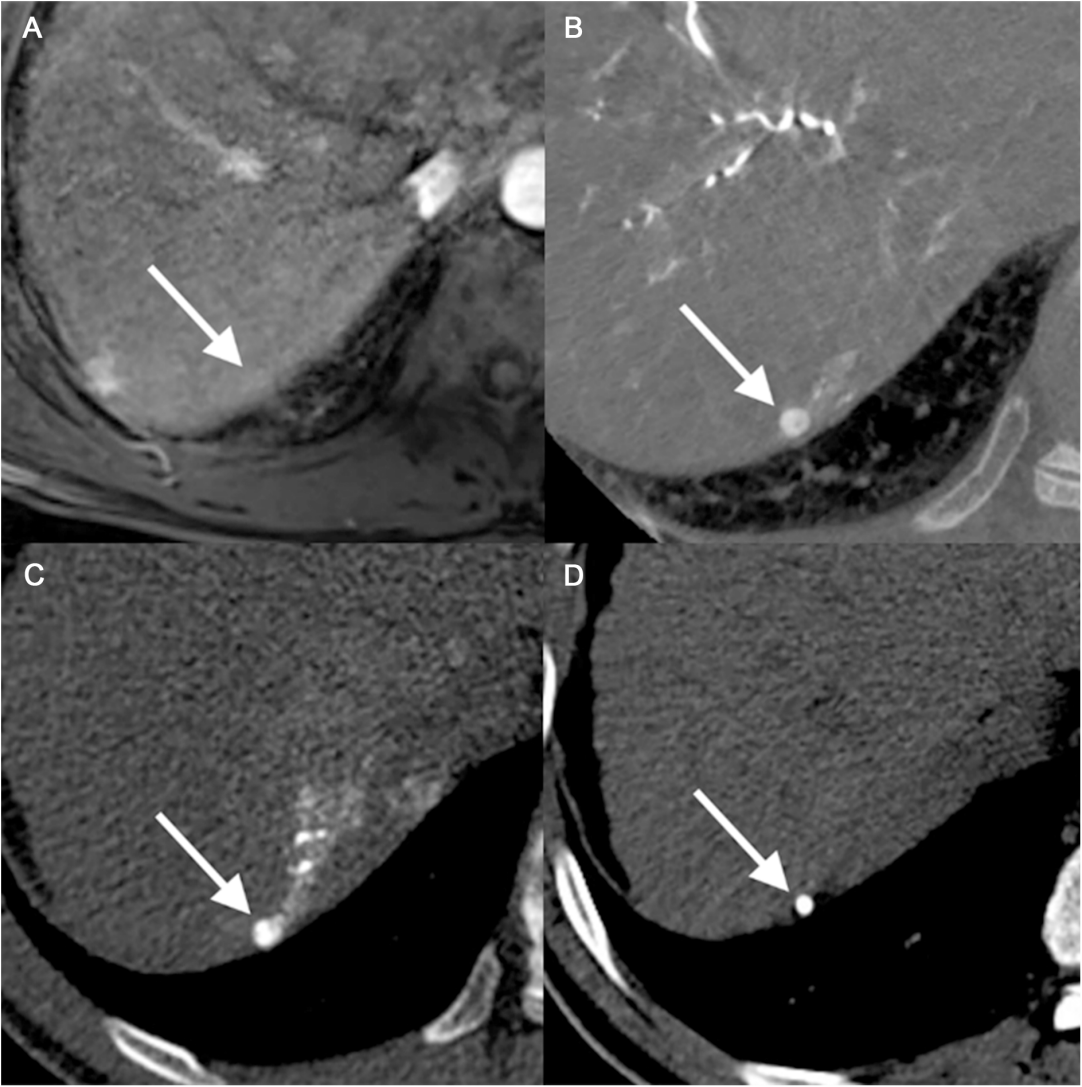
A 57-year-old man with small hypervascular HCC (Group 2). On initial MR, this nodule was indistinguishable from surrounding cirrhotic hepatic parenchyma on arterial phase (a), 3-minute delayed phase and hepatobiliary phase (not shown). (b) C-arm CT images show a 5mm-sized nodule (arrow) in the segment 7 dome of the liver as an enhancing nodule. (c-d) On noncontrast CT, compact lipiodol uptake of this nodule after TACE was noted (c) which was persistent after three months (d). None of the reviewers considered this lesion to be an HCC.

**Fig 5:**
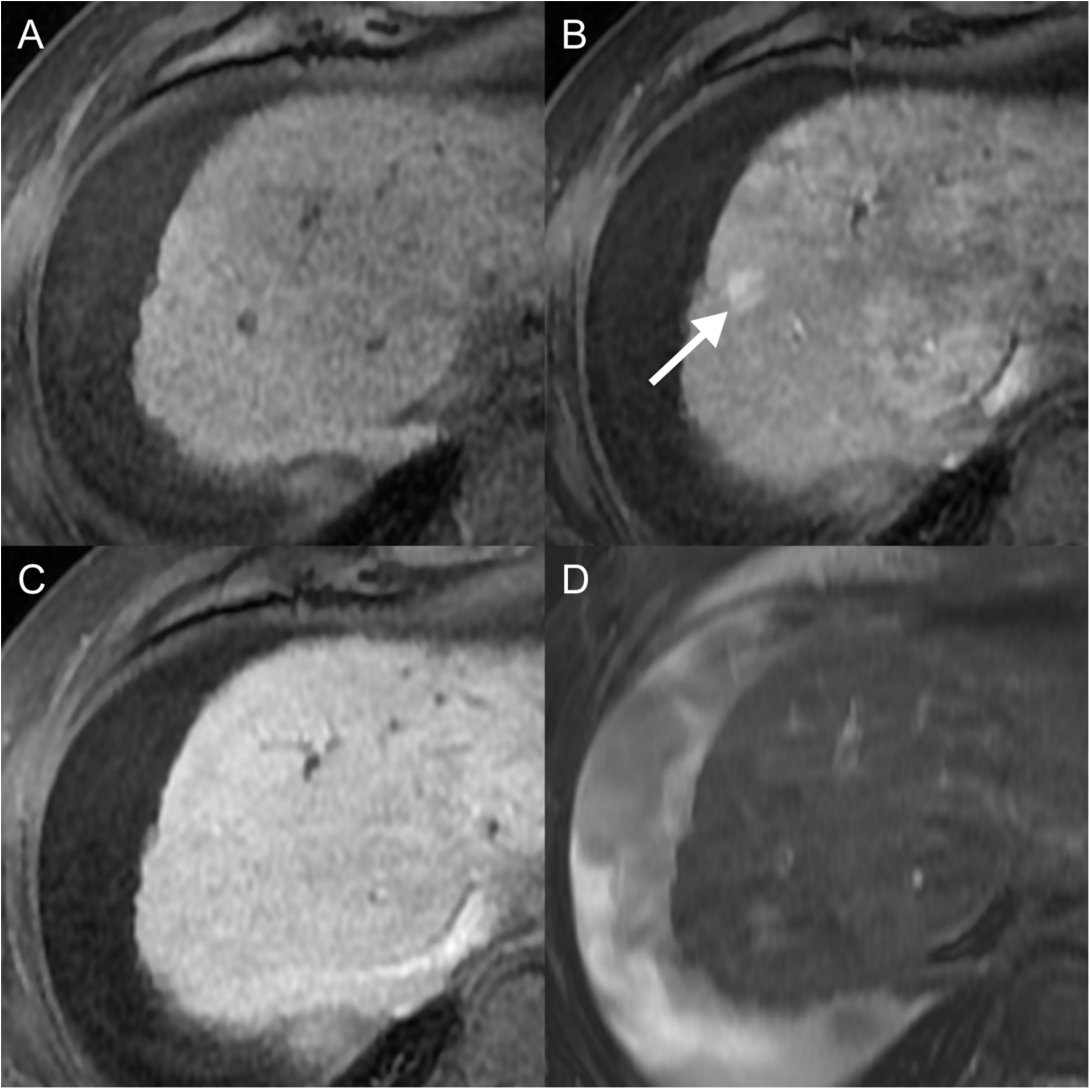
A 67-year-old man with a false positive lesion. There was an 11mm nodule with iso signal intensity on precontrast T1 weighted image (a) and hyperenhancement on arterial phase (b). However, this nodule did not show washout on delayed phase (c) or high signal intensity on T2 weighted image (d). This lesion did not uptake iodized oil after TACE (not shown). Although both reviewers graded this lesion HCC.

There were 10 large HCCs and 35 small HCCs that were not identified by any reviewer on MR images. Among the 35 small HCCs, ten lesions were too small (mean, 0.59 cm ± 0.17) to be detected on MR images (Fig 3). Four lesions were seen as nodules that showed only arterial enhancement (n = 1), hyperintensity on T2-weighted images without arterial enhancement (n = 1) or hypointensity on HBP without arterial enhancement (n = 2). The other four lesions were thought to have not been detected due to the presence of accompanying, multiple arterioportal shunts (n = 2) or to the location of the lesion (n = 2), i.e. one in the right hepatic dome and the other abutting another HCC. The remaining 17 lesions were not detected on gadoxetic acid–enhanced MR images even after careful inspection by the two abdominal radiologists who were given the information regarding the location and size of the HCCs (Fig 4). Regarding the large HCCs, nine lesions appeared as nodules showing only arterial enhancement (n = 3) or hypointensity on HBP without arterial enhancement (n = 6). The remaining lesion was thought to have been overlooked due to its close proximity to another HCC. The two reviewers detected 51 and 41, false-positive lesions on MR images, respectively. For the small HCCs, reviewer 1 detected 30 of the false-positive lesions and reviewer 2 detected 11 of these lesions. Most of these false-positive observations were identified as indeterminate nodules or nodular arterioportal shunts by reviewing the MR images (Fig 5). In the control group, the two reviewers found 5 and 10 false-positive lesions on MR, respectively. Again, most false-positive lesions in the control group were nodular arterioportal shunts or dysplastic nodules. Specificity was 92.4% and 84.8% for each reviewer.

The concordance rates of the HCCs according to the LI-RADS (LR) are summarized in **Table 4**. Among the 379 HCCs, 26% (99 of 379) met LR-5, 66% (259 of 379) met LR-4, and 1% (5 of 379) met LR-3. The remaining 16 HCCs were not appreciable on the initial MR images and were, therefore, excluded from the LI-RADS grading. LR-5 and LR-4 covered 94% (358/379) of the HCCs. Of the 257, large HCCs, 37% (96/257) and 63% (161/257) were assigned to LR-5 and -4, respectively. The MR imaging features of the LI-RADS of the small and large HCCs are summarized in **Table 5**. Common features of small HCC included arterial enhancement (81.9%, 100 of 122), HBP hypointensity (80.3%, 98 of 122), and delayed washout (72.9%, 89 of 122). All of the major features except for growth, HBP hypointensity, mild-moderate T2 hyper-intensity, restriction of diffusion, mosaic architecture, and intra-lesional fat were significantly more common in large HCCs than in small HCCs (*P* < .05).

**Table 4:** Number of the HCCs according to the LI-RADS.

		Arterial phase hypo- or iso- enhancement		Arterial phase hyper-enhancement		
Diameter (mm)		< 20	≥ 20	< 10	10–19	≥ 20
• Washout • Capsule • Growth	None:	LR-3 (n = 2)	LR-3 (n = 0)	LR-3 (n = 3)	LR-3 (n = 0)	LR-4 (n = 4)
	One:	LR-3 (n = 0)	LR-4 (n = 1)	LR-4 (n = 67)	LR-4 (n = 171)	LR-5 (n = 66)
					LR-5 (n = 2) if growth (+)	
	≥ Two:	LR-4 (n = 8)	LR-4 (n = 0)	LR-4 (n = 8)	LR-5 (n = 20)	LR-5 (n = 11)

**Table 5:** MR imaging features according to LI-RADS of Small (≤1-cm) and Large (>1-cm) HCC.

MR Imaging Features	Small (≤1-cm) HCC (n = 122)	Large (>1-cm) HCC (n = 257)	P Value†
Major features			
Arterial enhancement	100 (82)	252 (98)	<0.001
Delayed washout	89 (73)	238 (93)	<0.001
Capsule	5 (4)	28 (11)	0.028
Growth	23 (19)	4 (2)	<0.001
Ancillary features			
Hepatobiliary phase hypointensity	98 (80)	249 (97)	<0.001
Mild-moderate T2 hyper-intensity	50 (41)	185 (72)	<0.001
Restricted diffusion	45 (37)	165 (64)	<0.001
Corona enhancement	0 (0)	3 (1)	0.554
Mosaic architecture	23 (19)	143 (56)	<0.001
Nodule-in-nodule architecture	5 (4)	22 (9)	0.137
Intra-lesional fat	5 (4)	55 (21)	<0.001
Lesional iron sparing	0 (0)	0 (0)	
Lesional fat sparing	1 (1)	4 (2)	1.000
Blood products	0 (0)	2 (1)	1.000

Note.—Numbers in parentheses are percentages.

† P value was obtained from chi-square test or Fischer’s exact test of MR imaging features between small and large HCC.

## Discussion

In our study, we used JAFROC analysis which is resistant to clustering bias. Based on our results, the diagnostic accuracy of gadoxetic acid–enhanced MR imaging for small, hypervascular HCCs was as follows: a mean figure of merit of 0.787, mean sensitivity of 63.0% (153 of 244), and mean PPV of 79% (153 of 194). This result is lower than large HCCs with a mean figure of merit of 0.948, mean sensitivity = 91% (466 of 514), and mean PPV = 90% (466 of 517). Seventeen of 122 small HCCs (13.9%) were not visible on MR images, even after careful review. Arterial enhancement (81.9%), hepatobiliary phase hypointensity (80.3%), and delayed washout (72.9%) were common in small hypervascular HCCs. The concordance rate of the LI-RADS was 26% in LR-5 and 66% in LR-4.

The diagnosis of small HCCs is a subject of controversy. Many previous studies have reported that the findings of gadoxetic acid–enhanced MR imaging, such as diffusion-restriction, hypointensity at HBP, hyperintensity on T2-weighted images, and intra-lesional fat seen on dual-echo, T1-weighted MR images are useful for detecting small HCCs [21, 22]. However, according to some recent studies, the diagnostic performance for small HCC is still relatively low. A recent meta-analysis revealed that the sensitivities for lesions < 2 cm was only 55% for MR and Yu et al. reported 46.0% to 48.3% sensitivity for lesions ≤ 1 cm for gadoxetic acid-enhanced MR. [7, 8]. Compared with these results, our study yielded a relatively high sensitivity of 63.0% for small HCCs which could possibly be enhanced by the inclusion of only hypervascular HCCs. On the other hand, there have been studies that reported a higher sensitivity for small HCCs than that of our study [23, 24]. According to these reports, the mean value of the areas under the receiver operating curve were 0.869–0.952 and the mean sensitivity was 88.1% - 93.3%. This difference could have originated from the size of the HCCs studied as we included lesions with a diameter ≤ 1 cm, whereas Hwang et al. and Park et al. analyzed tumors which were < 2 cm in diameter. Because the difficulty in diagnosing HCCs on MR increases as the lesion size decreases, this is not a surprising result. Another possible explanation for this discrepancy is that some of the C-arm CT-aided detection of small hypervascular nodules had only subtle or no radiologic evidence of HCC on the initial MR.

In our study, imaging features frequently noted in small HCC seen on gadoxetic acid–enhanced MR included arterial enhancement (81.9%), HBP hypointensity (80.3%), and delayed washout (72.9%). These results correspond well with those found in earlier studies [8]. The high prevalence of arterial enhancement was in agreement with our expectation as we collected hypervascular HCCs using a referencing standard of C-arm CT. HBP hypointensity is one of the useful findings of gadoxetic acid-enhanced MR for diagnosing HCCs which usually demonstrate hypointensity on HBP due to the decreased or absent expression of organic anion transporter 8 [25]. Bashir et al. reported that HBP hypointensity improved the detection of small (≤ 1 cm) HCC in patients with underlying cirrhotic liver [26], and which is consistent with our findings. In the assessment of washout on the delayed phase, we selected portal-phase or transitional–phase images that yielded better contrast of the lesion compared to that of the surrounding hepatic parenchyma. According to a recent study by Joo et al. regarding washout seen on gadoxetic acid-enhanced MR, hypointensity on the portal phase and/or the transitional phase showed a lower specificity, although a higher sensitivity (86.3% and 86.6%, respectively) compared to that of the portal phase only (97.9% and 70.9%, respectively) [27]. Therefore, in our study, more HCCs would have been detected along with the inclusion of more false-negative lesions by including the transitional phase into the delayed phase compared with the portal phase only.

On the other hand, there was T2 hyperintensity in less than half of the small HCCs (41.0%), while approximately 72% of the large HCCs demonstrated this. This result strongly coincides with that of a previous report that a larger HCC was brighter on T2 weighted imaging than a smaller HCC [27]. There have also been studies that reported the usefulness of T2 weighted imaging in the detection of HCCs [5, 21], and mild-moderate T2 hyperintensity was recently adopted as an ancillary feature that may favor the diagnosis of HCC on LI-RADS [18].

Diffusion restriction was only observed in approximately one-third of the small HCCs (36.9%, 45 of 122). There have been studies which yielded opposing results regarding the role of DWI in the detection of small HCCs [9, 24, 28–30]. Park et al. reported improved sensitivity for detecting small HCCs by adding DWI to gadoxetic acid-enhanced, dynamic MR imaging and which increased the sensitivity from 80.5–82.1% to 91.1%—93.3% [24]. However, they included HCCs of a maximum diameter of 2 cm and less than one-third (55 of 179) of the lesions were ≤ 1 cm in diameter. The results reported by Le Moigne et al. in their study suggested that the diagnosis of small HCCs was improved by the addition of DWI to conventional dynamic MR imaging and which increased the sensitivity from 75.7% to 87.8%. However, as that study was also conducted on HCCs less than 2 cm in diameter their results may, therefore, may not apply to lesions ≤ 1 cm in diameter [29]. A few other studies have also demonstrated questionable results regarding DWI for detecting small HCCs, as in these studies conventional dynamic MR showed superior results to those of DWI or the addition of DWI had no diagnostic benefit [9, 30]. These unfavorable results of DWI regarding small HCCs could be explained by the following: as the tissue structure of HCC is similar to that of the surrounding cirrhotic liver, it can be difficult to differentiate an HCC from cirrhotic parenchyma [24]; the background cirrhotic liver parenchyma may show diffusion restriction [31]; and there is a limited signal-to-noise ratio and spatial resolution with susceptibility to motion artifacts [32]. Although DWI has the advantage of a short acquisition time, the aforementioned drawbacks of DWI would lower its diagnostic performance, especially when the lesion size is small.

In our study, the concordance rate of LI-RADS was 26% in LR-5 and 66% in LR-4. In total, 94% (358 of 379) of the HCCs were categorized as LR-4 or -5. Of the 257 large HCCs, 96 nodules and 161 nodules were assigned to LR-5 and -4, respectively. With regard to the 122 small HCCs, there were three LR-5 lesions and 98 LR-4 lesions. It is not surprising that there was 2% (3 of 122) LR-5 lesions in small HCCs because an observation smaller than 1 cm cannot be classified into LR-5. The other three LR-5 lesions in small HCCs was 1 cm in diameter. In addition, there was no LR-M (not specific for HCC), LR-5V (tumor in vein), LR-2 or LR-1 (probably or definitely benign, respectively) lesions in our results. Therefore, it can be said that LI-RADS demonstrated high sensitivity for HCC. To our knowledge, there has been only one previously published study that utilized LI-RADS in the analysis of HCCs [33]. However, this study focused mainly on the usefulness of HBP imaging in the LI-RADS and was conducted including only a limited number of lesions.

There are some limitations to our study. First, the use of C-arm CT as a standard of reference has not been clinically validated despite the fact that there have been previous studies advocating the role of C-arm CT in the detection of HCCs [8, 34]. There is also no diagnostic criterion for small (≤ 1 cm) HCCs according to AASLD and EASL [2, 3]. The pathologic diagnosis, which is a traditional gold standard in the diagnosis of HCC, also has limitations in that the section interval of the total hepatectomy specimens being used at our hospital is usually 0.5 cm or 1 cm. Considering this limitation in the selection of the referencing standard for small HCCs, we used additional criteria other than hypervascularity on C-arm CT so as to increase the reliability of our results. The growth seen on follow-up, C-arm CT with diagnostic imaging features on CT or MR or continuous, iodized-oil uptake after TACE were required for a lesion to be regarded as an HCC, and as Yu et al. previously described [8]. Second, as ours is a retrospective study, MR units of multiple vendors with concomitant differences in sequence and image acquisition parameters, such as TR or TE, could have affected the image quality. Moreover, some sequences, including DWI or fat-suppression imaging, was not performed in all patients. Third, inclusion of the patients who had undergone treatment for HCC, such as radiofrequency ablation or TACE, might have caused difficulty in the interpretation by the reviewers. Detection of HCCs can be challenging in the region adjacent to post-treatment change, especially for small HCCs. However, at the same time, because it is close to real practices, our results could more applicable to the clinical practices. Last, the target population of our study was candidates for TACE. Therefore the lesions were likely to be hypervascular and multiple. It may not be generalized in the other population with isovascular or hypovascular lesions.

In conclusion, the diagnostic accuracy of gadoxetic acid–enhanced MR imaging for small, hypervascular HCCs (Mean figure of merit = 0.787) was still low compared with large HCC (Mean figure of merit = 0.948). LR-5 and LR-4 covered 94% (358/379) of the HCCs.

## Supporting information

S1 Table Pulse sequence parameters of gadoxetic acid–enhanced MR images. (DOCX)
