# Supplementary material for: Diagnostic accuracy of gadoxetic acid-enhanced MR for small hypervascular hepatocellular carcinoma and the concordance rate of Liver Imaging Reporting and Data System (LI-RADS)
Source: PLoS One. 2017 May 30;12(5):e0178495. doi: 10.1371/journal.pone.0178495 (PMC5448778; doi:10.1371/journal.pone.0178495)
Supplement: S1 Table — (DOCX) [file pone.0178495.s001.docx]

**S1 Table.** **Pulse Sequence Parameters of Gadoxetic Acid–enhanced MR Images.**

| **MR Unit No.** | **Fat-saturated T2-weighted Sequence** | | | | | **Dynamic Three-dimensional Fat-saturated T1-weighted Sequence** | | | | | **DWI** | | | | |
| --- | --- | --- | --- | --- | --- | --- | --- | --- | --- | --- | --- | --- | --- | --- | --- |
|  | TR/TE | Flip Angle  (degrees) | Section  Thickness  (mm) | Field of View | Matrix | TR/TE | Flip Angle  (degrees) | Section  Thickness  (mm) | Field of View | Matrix | TR/TE | Flip Angle  (degrees) | Section  Thickness  (mm) | Field of View | Matrix |
| **1 (1.5T) *** | 2000/84 | 90 | 7 | 359×359 | 256×256 | 5.0/2.2 | 12 | 6 | 350×350 | 320×224 | 6500/61 | 90 | 7 | 400×400 | 128×96 |
| **2 (3.0T)†** | 3898/95 | 140 | 7 | 379×308 | 448×2 | 3.4/1.2 | 11 | 3 | 379×308 | 384×250 | 4500/56 | 180 | 8 | 379×379 | 140×140 |
| **3 (3.0T)‡** | 3000/92 | 140 | 7 | 380×380 | 320×320 | 3.3/1.2 | 11 | 3 | 379×308 | 384×250 | 4300/59 | 180 | 8 | 379×379 | 140×140 |
| **4 (3.0T)§** | 1789/160 | 90 | 7 | 380×380 | 332×291 | 3.3/1.6 | 10 | 6 | 379×379 | 344×277 | 1321/66 | 90 | 7 | 399×399 | 128×102 |

Note.—TR/TE = repetition time (msec)/echo time (msec).

* Signa HDx, GE Healthcare.

†Verio, Siemens Healthcare

‡Trio, Siemens Healthcare.

§Ingenia, Philips Medical Systems
